# An actionable understanding of societal transitions: the X-curve framework

**DOI:** 10.1007/s11625-021-01084-w

**Published:** 2022-01-18

**Authors:** Aniek Hebinck, Gijs Diercks, Timo von Wirth, P. J. Beers, Lisa Barsties, Sophie Buchel, Rachel Greer, Frank van Steenbergen, Derk Loorbach

**Affiliations:** 1 Dutch Research Institute for Transitions (DRIFT), Erasmus University Rotterdam, Rotterdam, The Netherlands; 2 Erasmus School of Social and Behavioural Sciences (ESSB), Erasmus University Rotterdam, Rotterdam, The Netherlands; 3 HAS University of Applied Sciences, ‘s Hertogenbosch, The Netherlands; 4 Department of Obstetrics and Gynaecology, Erasmus MC, University Medical Centre Rotterdam, Rotterdam, The Netherlands

**Keywords:** Societal transitions, Action research, Transition dynamics, Build-up, Breakdown, Transformative governance

## Abstract

Sustainability transition research seeks to understand the patterns and dynamics of structural societal change as well as unearth strategies for governance. However, existing frameworks emphasize innovation and build-up over exnovation and break-down. This limits their potential in making sense of the turbulent and chaotic dynamics of current transition-in-the-making. Addressing this gap, our paper elaborates on the development and use of the X-curve framework. The X-curve provides a simplified depiction of transitions that explicitly captures the patterns of build-up, breakdown, and their interactions. Using three cases, we illustrate the X-curve’s main strength as a framework that can support groups of people to develop a shared understanding of the dynamics in transitions-in-the-making. This helps them reflect upon their roles, potential influence, and the needed capacities for desired transitions. We discuss some challenges in using the X-curve framework, such as participants’ grasp of ‘chaos’, and provide suggestions on how to address these challenges and strengthen the frameworks’ ability to support understanding and navigation of transition dynamics. We conclude by summarizing its main strength and invite the reader to use it, reflect on it, build on it, and judge its value for action research on sustainability transitions themselves.

**Supplementary Information:**

The online version contains supplementary material available at 10.1007/s11625-021-01084-w.

## Introduction

Transition research seeks to understand the dynamics of change and to find leverage for guiding societal transitions (Grin et al. 2010; Loorbach et al. 2017). It presents societal transitions as the structural and systematic reconfiguration of a stable societal system towards a new dynamic equilibrium, understood to evolve over decades or generations (Geels and Schot 2007; Grin et al. 2010; Kanger and Schot 2019). This body of research has been vital for better understanding of past transitions; it has supported the identification of the underlying patterns and mechanisms driving transformative system change and has been instrumental in exploring the role of agency in driving processes of change. In this, two frameworks were highly influential: the ‘multi-level perspective’ aimed at understanding long-term and complex socio-technical transitions^1^ (Rip and Kemp 1998) and the ‘multi-phase’ concept, describing transition dynamics as alternating phases differing in speed, size, and timing^2^ (Rotmans et al. 2001). These frameworks share the pattern of emerging and diffusing niches. The multi-level perspective emphasises the interaction of niches with the socio-technical regime and the underlying dynamics of structure and agency through markets, industry, science, policy, technology, and culture. The multi-phase concept, rooted in models of demographic change and innovation diffusion, emphasises non-linearity and presents a narrative of frontrunners and change agents in driving transformative change (Notestein 1948; Rogers 1995; Rotmans et al. 2001).

These existing frameworks have contributed key insights into societal transitions. However, when it comes to their explanatory value for transitions-in-the-making, we believe these frameworks have become inapt as they consistently emphasise the dynamics of alternative build-up and innovation (Heyen et al. 2017; Loorbach et al. 2017; Davidson 2019). They fall short in making sense of current dynamics of change that involve both build-up and breakdown patterns, which prove to be turbulent, chaotic, and unstructured (Carpenter et al. 2019). This is troublesome, as research finds people are biased towards novelty in solving problems and systematically overlook subtractive changes (Adams et al. 2021). As such, it is important to make processes of decline, breakdown, and phase-out more explicit in frameworks that describe the dynamics of societal change.

There are several existing frameworks addressing breakdown dynamics, but their insights have mostly remained conceptual and thus difficult to translate to actionable governance measures. For instance, the ‘panarchy cycle’ (Holling 2001; Gunderson and Holling 2002) is grounded in resilience thinking and describes unpredictable cycles of both renewal and collapse in ecosystems, but says less about agency for change (Allen et al. 2014). Or, institutional failure literature understands crisis as a source for change, or even a window of opportunity, but does not systematically assess in which ways or under which conditions this might take place (Derwort et al. 2019). To support actors in navigating societal transitions, there is a need for complementary perspectives that integrate the build-up and stabilisation of alternatives as well as the destabilisation and decline of existing social practices and structures to comprehend and guide the turbulences of transitions-in-the-making.

This paper addresses this gap by expanding on the development and use of the X-curve framework. This framework offers actionable support in understanding and developing governance practices for sustainability transitions by revealing interactions between the dynamics of build-up and breakdown. We follow Sharpe et al. (2016) in presenting our work as a ‘practice note’: we do not set out to present this work as an ‘academic piece’ that tests the framework’s effectiveness or presents evidence of concrete impacts. Instead, we focus on articulating the co-creative and iterative development process of the X-curve framework and illustrate its use in various settings, with the intention for others to use, reflect, judge, and build on the X-curve framework. We start by showing how a societal demand for new knowledge spurred thinking about breakdown and marked the starting point of the development of the X-curve as an approach to structure actionable thinking in an applied setting. We continue by illustrating the use of the X-curve framework and how it reveals a set of typical dynamics in diverse settings. We discuss challenges in using the framework and provide suggestions on how to address these challenges and strengthen the frameworks’ ability to support understanding and navigation of transition dynamics. We conclude by summarizing its main strength and invite the reader to use it, reflect on it, build on it, and judge its value for action research on sustainability transitions themselves.

## A societal demand for knowledge on breakdown

Thinking about exnovation and phase-out was spurred by societal incidents and developments that highlighted the limits of particular systems. For example, the nuclear accident at Fukushima, Japan (2011), was a disruptive event and early driver for phase-out plans of nuclear energy in Germany and key to the debate on energy transitions (Kramm 2012). In The Netherlands concerns about earthquakes (2012) sparked debate about phasing out natural gas extraction in Groningen (Oxenaar and Bosman 2019). Such events made the need to consider regime destabilisation and phase-out increasingly self-evident and brought it to the core of policy discussions. This, in turn, raised expectations of science to deliver knowledge that addresses these challenges and that fits policymakers’ needs to design policy mixes concerning phase-out strategies (Dilling and Carmen 2011; Kirchhoff et al. 2013).

Work that engages with destabilisation, phase-out, and exnovation more explicitly has tried to understand how a combination of factors can make it increasingly conceivable and likely that an unsustainable regime will destabilise and structurally reorganise, creating space for alternatives to emerge, leading to reconfiguration and a disruptive shift towards a new equilibrium. For instance, work on system change dynamics conceptualises destabilisation dynamics as facets of a ‘breakdown trajectory’ (Turnheim and Geels 2012; Markard 2018; Davidson 2019). More commonly, this is referred to as a process of ‘exnovation’, which describes a long-term process during which an existing practice or socio-technical configuration is phased out and stepwise removed. Here, phasing-out is based on deliberate decisions, which distinguishes the idea of exnovation from concepts such as ‘discontinued use’ (David 2017; Heyen et al. 2017; Davidson 2019). Processes of exnovation are moving into focus when existing socio-technological configurations are increasingly under pressure by being ‘societally framed as obsolete and undesirable, particularly in regard to their environmental externalities’ (David 2017). In addition, transition scholars have also started to address these dynamics in the context of identifying appropriate policy mixes to instigate and accelerate both the institutionalisation of more sustainable alternatives and the de-institutionalisation and phase-out of existing unsustainable practices (Kivimaa and Kern 2016; David 2017; Greer et al. 2020; Oers et al. 2021).

Notions such as destabilisation and phase-out have also become more evident in the consultancy projects and action research conducted by DRIFT researchers and advisors^3^ since 2001. From the onset, these applied (research) projects built on a transition management^4^ approach and often entailed engaging with policymakers that were part of the regime. As societal events such as the earthquakes in Groningen unfolded, issues relating to systemic destabilisation increasingly came to the fore in these exchanges. While much of the transition-related research had focussed on these change dynamics in a more isolated sense, what was missing was a systemic and actionable understanding that could guide governance and users in an applied setting. As such, the existing frameworks became increasingly unfit to guide sustainability governance of transitions-in-the-making in an applied setting and were unable to respond to this demand for new knowledge. This led to the development of new frameworks, which involved combining the existing and emerging transition frameworks with insights and frameworks from outside the transition network, with the aim of meeting practical needs regarding strategic insights into destabilisation and phase-out dynamics. The first iteration towards the X-curve framework was explicitly driven by this motivation and aimed to do so through action-oriented knowledge creation. In the process of making sense of these new developments in a transition management context and responding to the demand for new knowledge, the X-curve iteratively came about over the last decade (Loorbach 2014; Lodder et al. 2017; Bode et al. 2019).

### Iterations towards the X-curve

**Fig. 1:**
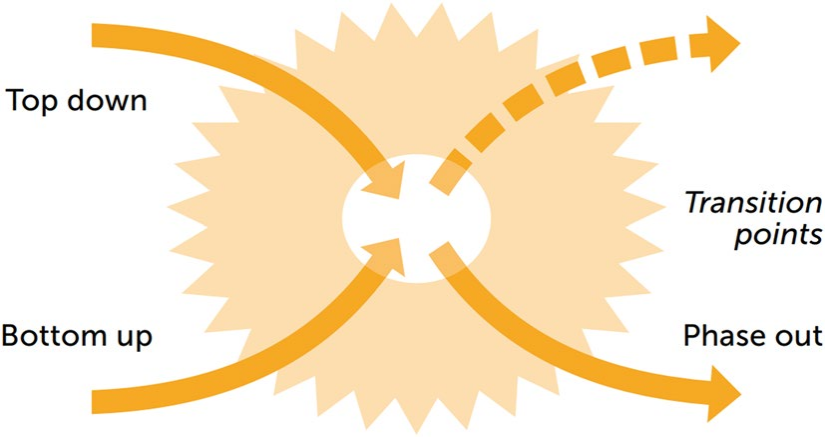
Earlier version of the X-curve that focussed on the inclusion of system collapse from Loorbach (2014)

The first version of an X-curve in Loorbach (2014) captured the X-shaped pattern of build-up and phase-out while linking it directly to agency and governance. This was in reaction to the increasing relevance of breakdown dynamics for transitions-in-the-making to various societal actors, leading to the combination of earlier developed transition frameworks with the analytical insights of breakdown dynamics such as exnovation. This included three quadrants and identified these as ‘top-down’, ‘bottom-up’, and ‘phase out’, leaving the upper right quadrant open (Fig. 1). This first framework’s inclusion of the collapse of a system provided a basis to shift the focus in policy discussions from innovation to phase-out and on using the government’s top-down role to instigate and accelerate transitions. However, soon it became clear that this first version (Fig. 1) did not have a sufficient analytical basis for practical and actionable use in policy discussions, as users required more detailed insights into the change dynamics at play.

**Fig. 2:**
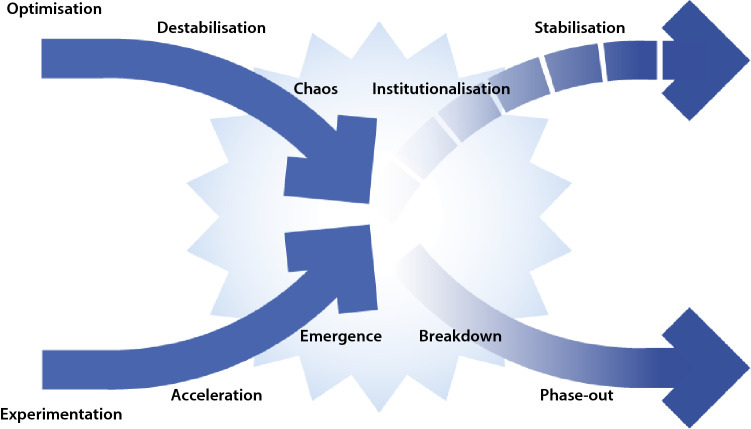
The X-curve portraying the interaction of patterns of build-up and breakdown (based on Loorbach et al. 2017)

To identify more detailed dynamics that play a role in the patterns of breakdown and build-up, we have built on expertise in socio-technical, socio-institutional, and social-ecological system change (Loorbach et al. 2017). Several important sources were included in the conceptual development. First, the emerging insights into niche-regime interactions in transition studies, which emphasised the need for ‘a less hierarchical representation’ of transitions (Diaz et al. 2013, p. 72; Smink et al. 2015; Hess 2016). Second, the theoretical underpinning of the framework drew on institutional theory’s understanding of institutional and policy failure (Scott 2013; Derwort et al. 2019), leading to structure-related insights into destabilisation and (re-)institutionalisation (Turnheim and Geels 2013; Fuenfschilling and Truffer 2014). Third, the framework builds on seminal work in innovation theory, for example, Schumpeterian assumptions about creative destruction, described as an ongoing mutation process that inherently revolutionises the industrial (or economic) structures from within. This continued renewal simultaneously includes the destruction of existing structures and the creation of new ones (Schumpeter 1942). The X-curve framework also builds upon theory from social-ecological system studies, such as integrating the notions of chaos, emergence, and co-evolution, which are originally rooted in complexity theory (Prigogine and Stengers 1984; Holland 1995; Kauffman 1996). These complex change dynamics are also conceptualised in the ‘panarchy cycle’, which shows the inherent and continuous dynamics of collapse and renewal in ecological systems through processes of decomposition and redefinition (Holling 2001; Gunderson and Holling 2002; Allen et al. 2014). This, in particular, has been influential for the X-curve’s focus on chaos and conflict as a functional space. Lastly, broader work from the resilience community has informed an understanding of transitions as non-linear and tending towards an equilibrium state (Walker et al. 2004). These existing insights from varied scientific literature provide an understanding of more specific dynamics of change and have been combined in a simple visual framework, the X-curve (see Fig. 2).

The resulting X-curve framework was tested in applied contexts that had the aim of supporting practitioners in making sense of (changing) transition dynamics and allowing them to reflect on their own role and agency in these dynamics. A key co-creative moment to evaluate the X-curve framework was a project commissioned by the Dutch Ministry of Infrastructure and the Environment, which entailed the use of the X-curve thinking to map the state of transition and its policy implications for several key sustainability challenges relevant for the ministry^5^ (Lodder et al. 2017). This project allowed for the evaluation of the more detailed X-curve framework as an analytical tool for assessing transition dynamics, but also refinement of the framework as a sense-making device in a participatory setting. For instance, it provided concrete insights into how the patterns of build-up and breakdown and their underlying dynamics were experienced (see Table 1). The application of the framework in this project also confirmed the framework’s ability to facilitate dialogue between researchers and practitioners and to identify a joint basis for action.

**Table 1:** Patterns of build-up and breakdown and underlying dynamics (Lodder et al. 2017, translated from Dutch)

Pattern	Of build-up	Pattern	Of breakdown
Experimentation	Radical new practices Radical new thinking	Optimisation	Improving the existing No doubts about the system
Acceleration	Alternatives are connecting Alternatives are visible and accessible	Destabilisation	Incidents lead to (sense of) urgency Fundamental discussions about desired the direction
Emergence	New structures become visible Need for transition is broadly accepted	Chaos	Contradictions and uncertainties Opposing interests and conflict
Institutionalisation	The new normal (thinking and doing) Solidifying new structures	Breakdown	Repelling and releasing former established order Losers of processes of change become visible
Stabilisation	Tweaking Optimising	Phase-out	Saying goodbye Dealing with loss

## X-curve: transition dynamics of build-up and breakdown

In this section, we present the X-curve, as it is currently applied in both research and practice at DRIFT. In its essence, the X-curve framework presents a stylised visualisation of transition dynamics emphasising the interaction of build-up and breakdown dynamics that are associated with transitions (Loorbach 2014). It identifies ten different, yet typical, transition dynamics. However, these categories should not be seen as mutually exclusive or objectively quantified. Rather, they provide a starting point to explore the transition dynamics present in a given sector or system, and how patterns of build-up and breakdown might interact (see Fig. 2 and Table 1).

The identified dynamics build on various theories, amongst others (see the section ‘Iterations towards the X-curve’), earlier transition theory (Kemp et al. 1998; Geels 2005), on the notions of ‘creative destruction’ and exnovation (Schumpeter 1942; Davidson 2019), and the panarchy cycle (Gunderson and Holling 2002; Allen et al. 2014). It asserts that transitions always involve two central dynamics: one of ‘creation’, which builds and institutionalises new, alternative practices and structures, and one of ‘destruction’, which refers to the destabilisation and breaking down of existing practices and structures. The X-curve framework captures the interactions between patterns of build-up and breakdown and does so by focussing on the varying roles ‘regime’ and ‘niche’ can play in this.

### Pattern of breakdown

As the regime is increasingly confronted with pressures to adapt and optimise to maintain its (dynamic) stability (Geels and Schot 2007), it will increasingly become incongruent, leading to ‘enhanced lock-in’ and path dependence (Unruh 2002). Continued efforts and investments for *optimisation* of the regime then make it more difficult to change fundamentally (Geels 2011, 2014; Turnheim and Geels 2012, 2013; Kuokkanen et al. 2018). Under continued exogenous pressure, this leads to *destabilisation*, which is associated with unrest, tensions, and emerging doubts (Turnheim and Geels 2012; Kuokkanen et al. 2018; Leipprand and Flachsland 2018; Carpenter et al. 2019).

Destabilisation can be the starting point for a dynamic framed in the X-curve as ‘chaos’. This is a phase of a sudden loss of security, collapse of stable institutions and established organisations, and profound political interventions or acute crises (Markard 2018). *Chaos*, as used here, has its origins in complexity theory (Prigogine and Stengers 1984; Holland 1995; Kauffman 1996) and represents the state of a system that is out-of-equilibrium, and future directions and configurations of the equilibrium-to-be are both uncertain and ambiguous. Ultimately, this chaos leads the regime structure to be unable to fulfil its function, eventually leading to the breakdown and phasing-out of (parts of) the original elements of the regime (Rogge and Johnstone 2017; Oei et al. 2020).

### Pattern of build-up

Interacting with the dynamics of breakdown dynamics is a pattern of build-up dynamics. Dynamics of build-up describe the ‘transformative innovations’ that often arise through *experimentation* with radical new practices and thinking that are shielded from the pressures of the regime (Smith and Seyfang 2007; Sengers et al. 2019). These innovations are considered radical, alternative, marginalised, outside the social norm, and a reaction to the dominant way of thinking and doing (Smith et al. 2015; Avelino et al. 2019). Over time, these alternatives can become cheaper, more visible, better understood, and self-organised, contributing to *accelerating* processes of diffusion. Diffusion generally entails some level of spreading, scaling, and translating (von Wirth et al. 2019; Loorbach et al. 2020), as a growing number of people support the innovation and doubts about the long-term feasibility of the regime grow.

Gradually, processes of diffusion and the self-organisational capacity of people allow for new structures, routines, and organisational forms to *emerge* as ‘niche-regimes’ (Holland 1992; de Haan and Rotmans 2011; Diaz et al. 2013). Niche-regimes then lead to *institutionalisation* as actors from within the regime start to engage with the transition, leading to the creation of new alliances, routines, norms, and cultures by combining elements of both niche and regime (Kemp et al. 1998; Raven et al. 2010; Fressoli et al. 2014; Pel 2016). This gradually results in the *stabilisation* of a new regime.

Central to the useability and legitimacy of the X-curve framework is the perspective that transitions are a subjective notion, depending upon the observer’s viewpoint and their contextual assessment of transition dynamics. For example, what is considered to be the dominant ‘regime’ or alternatives are subject to deliberation: depending on preferences, objectives, or perceived path-dependencies, different assessments of current and anticipated transition dynamics can be made. As such, the X-curve combines insights from across transition research into a single framework for collective transition analyses and sense-making.

## Reflections on using the X-curve in practice

This ‘practice note’ (Sharpe et al. 2016) came about through a post hoc qualitative reflection of three cases, which allowed us to reflect on how and to what extent the X-curve framework enabled the creation of action-oriented knowledge. These cases are the outcome of action research, and this paper is based on the experiences of the authors of this paper. Together, they entail several years of experience in using the X-curve in interactive sessions to generate action-oriented knowledge. Experiences are drawn from different contexts, ranging from education, consultancy, and research. This paper is intended to provide concrete insights into the use of the framework, intending to lay a foundation for the use of and reflection on the X-curve by other practitioners and action researchers.

All cases were projects carried out and facilitated by the institute to which the authors are affiliated. The three cases analysed in this paper were selected based on a diversity of contexts to display the versatility of the framework. The cases differed contextually in terms of sustainability challenges, scale, societal setting, and participants (see Table 2). Nevertheless, they were alike in the following ways: they explicitly featured one or more interactive sessions in which the X-curve was used as a framework. They all used the same depiction of the X-curve during the respective sessions. These three cases cover research, advisory, and education activities, which generated data in several ways (see the Supplementary Material for an in-depth description of the cases). During the X-curve sessions, stakeholders (or participants) would use sticky notes, pens, markers, or a digital Miro board and occasionally pictures to share their ideas regarding the transition challenge at hand. This would feed into developing an X-curve diagram, filled out with various transition dynamics, serving as a shared representation of the group’s collective thought process. Second, the proceedings of each session would be further reflected and be summarised either in the form of a session report or as part of a broader advisory document. Both primary and secondary data were revisited to illustrate the use of the X-curve in practice and reflect on its strengths and weaknesses in creating actionable knowledge. Tables and figures from these projects are used as means of illustration.

**Table 2:** The three cases and their contextual differences

	‘University minor’	‘Healthy pregnancy project’	‘Ministry advisory work’
Setting	An interdisciplinary university minor on ‘new economic thinking’	A research project on how to reduce perinatal health inequities	Advisory work for the Dutch Ministry of Infrastructure and Water
Scale	Exercise on global food system dynamics involving 45 students	6 Dutch municipalities across the Netherlands	3 directorates within the ministry
Sustainability challenge	Creating awareness and educating about the change dynamics and envisioned transition futures of the food system	Identify and develop transformative change towards the implementation of perinatal health into municipal approaches and policies concerning health inequities	Assessing the state of transition with respect to mobility, circular economy, and climate adaptation in the built environment
Workshop participants	Bachelor students at the Erasmus University Rotterdam	Professionals working in the medical, social, or public health sector or for the municipal government	Civil servants from the three directorates: sustainable mobility; sustainable environment and circular economy; and water safety, climate adaptation, and governance

In this section, we use these cases to illustrate how the X-curve can help enable the creation of action-oriented knowledge. We understand action-oriented knowledge creation as a co-creation process between diverse societal actors that lead to new knowledge and capacities of involved actors. The work by Schneider et al. (2019) builds on ProClim and CASS (1997) and outlines three categories of knowledge that each differently supports actions for sustainability transitions: first, *systems knowledge*, referring to knowledge about the ‘what’ and its descriptive and explanatory aspects about the current system state; second, *normative knowledge* about the desired goals and ‘what should be’ or ‘what could be’, allowing room for elements such as (contested) norms and values related to the desired future development; and third, *transformation knowledge* about ‘how’ we may come from current states to where we should be, addressing actions, pathways and governance conditions for change, as well as concrete measures and in-between targets to put transformation into action.

Although all cases display elements of all three knowledge categories, we find that each case has shown particular strength in one specific category. We therefore use each case to illustrate one category of knowledge creation.

### ‘University minor’: strengthening system knowledge

In the case of the ‘*university minor*’, our use of the X-curve aimed to teach bachelor students to perceive system change from a comprehensive point of view, as well as gaining an understanding of diverse transition dynamics. During this exercise, a group of 45 students addressed long-term change processes in the global food system. Students received a one-hour introduction lecture on transition dynamics before this exercise, including the X-curve framework. In groups of five, students then collected answers to the four guiding questions. First, the future sustainability vision of a global food system was discussed with the question: ‘*what are the guiding principles of a radical transition future of a sustainable global food system?*’ Aspects that students collected were, for example, fair and true pricing for agricultural products, a strong focus on local production and sourcing, a substantial increase in healthy food education, minimised food waste, or taxation of meat consumption. In a second step, the X-curve exercise continued with a reflection on system characteristics that are undesirable, should be phased out, and should be broken down. Here, participants mentioned, for instance, the following aspects: agricultural monocultures, single-use plastic packaging, import dependencies, traditional meat-focussed diets, industrialised farming, or year-round accessibility of global food produce. Students then turned to emerging alternatives and discussed transformative innovations that should be further built up within the food system. Some examples of the responses provided by students were food banks, urban (rooftop) farming, circular food economy, hydroponics and floating farms, novel food taxation, full organic small-scale farming, and seasonal consumption. Finally, students focussed on what aspects of the existing food system to modify but continue, answering the question: ‘*what do we need to keep yet change in the current system and how can we adapt or modify to make this transition happen*? Here, students mentioned aspects such as increasing meat prices and finding incentives for vegetarian diets. When re-convening after this group work, there was a plenary reflection on the gained insights, the usefulness of the framework, and aspects that were overlooked.

Overall, the X-curve exercise supported the development of a comprehensive overview of a wide variety of transition dynamics, creating awareness about desired future visions and the emerging alternatives, as well as the current dominant practices that should be either converted or phased out. The juxtaposition of build-up and breakdown dynamics in a single figure appears to be key in allowing participants to reflect, evaluate, and define both desired and undesired systems in a comprehensive fashion.

### ‘Ministry advisory work’: facilitating normative knowledge

In addition to strengthening system knowledge, the X-curve is a useful framework to facilitate normative knowledge. This is mainly because of its ability to allow for different perspectives within the same framework (i.e., someone can perceive an event as experimentation, whereas another participant might interpret this as optimisation), offering a foundation for dialogue on what is the observed transition dynamic and what might be priorities that can be derived from that. There is an explicit need for the definition of the desired future system, as this provides (normative) direction to sustainability transitions and a foundation for both interpreting transition dynamics and derived strategic interventions.

The ‘*ministry advisory work*’ case illustrates this facilitation of normative knowledge in several ways. In interactive sessions, we started by co-producing an explicit version of the desired future. The discussion started by reflecting on the relevant system’s boundaries and underlying assumptions. For mobility, this resulted in the explicit inclusion of—and preference for—public transport, cycling, and walking. It resulted in desired future visions that questioned the dominance of individual car-based mobility and underscored the need for inclusivity and accessibility to prevent ‘mobility poverty’. This contributed to a broadening of the transition horizon towards systemic change, which in turn made radical visions more explicit, focussing on transforming to a sustainable and just mobility system (see Table 3). This prevented visions guided by a singular focus on technological solutions for current problems (i.e., substitutions of combustion engines for electric engines).

**Table 3:** Output from the ‘*Ministry Advisory Work*’: the interpretation of the current mobility transition dynamics, based on a combination of expert interviews, desk-research, and input from co-creation sessions with civil servants

Pattern	Of build-up	Pattern	Of breakdown
Experimentation	Pilots with mobility as a service and mobility hubs, new sustainable fuels (synfuels, biofuels, hydrogen), hyperloop, self-driving vehicles, mobility cooperatives	Optimisation	Mixing fossil fuels with biofuels; smart mobility to improve flow-through of car traffic; huge investment in road infrastructure maintenance and expansion; continuing rise of car ownership and airplane kilometres
Acceleration	Shared mobility concepts (bicycles, e-scooters, cars)	Destabilisation	Increasing congestion and overcrowded trains; increasing public debate about airports and ‘flight shame’; EU climate policy
Emergence	Electric cars, streets/city centres designed around bicycles instead of cars, e-bikes	Chaos	Sudden implementation of new maximum speed (from 130 to 100 km/h), court ruling limiting nitrogen emission
Institutionalisation	Modest signs, especially in establishing direction and ambitions in policy plans	Breakdown	Decreasing parking norms in residential areas
Stabilisation	Not identified	Phase-out	National government plans to ban the sale of new fossil fuel cars in 2030; the City of Amsterdam plans to impose an emission-free city centre in 2030

For the topic of circular economy, the X-curve highlighted the stark difference and complex interactions between the ‘old’ circular economy focussing on efficiency and recycling and the ‘new’ circular economy focussing on design and new business models. This led to a reflection by the civil servants on their own role in these parallel phases and an explicit interrogation and questioning of the existing institutions of the waste regime. Transition dynamics such as optimisation and destabilisation provided an intuitive way to discuss concepts such as path-dependency and lock-in and their own role in this. For instance, it made explicit that innovations in the recycling industry could still feed into optimisation (e.g., a technology that increases the efficiency with which to convert waste into energy), therefore enhancing lock-in rather than alleviating it. In sum, the use of the X-curve for the guiding of normative knowledge was best illustrated through the ‘*ministry advisory work*’ case. It helped with (self-) reflection on hegemonic positions and whether broader trajectories of change were still aligned with those of the imagined and desired sustainability transition.

### ‘Healthy pregnancy project’: guiding transformative knowledge

Building on these contextualised learnings about the system and its desired sustainability direction, the X-curve can then feature as a framework to identify transformative knowledge. Based on the X-curve-induced learnings about what transition dynamics are required to be strengthened or weakened, participants can then use the framework to identify potential interventions.

**Fig. 3:**
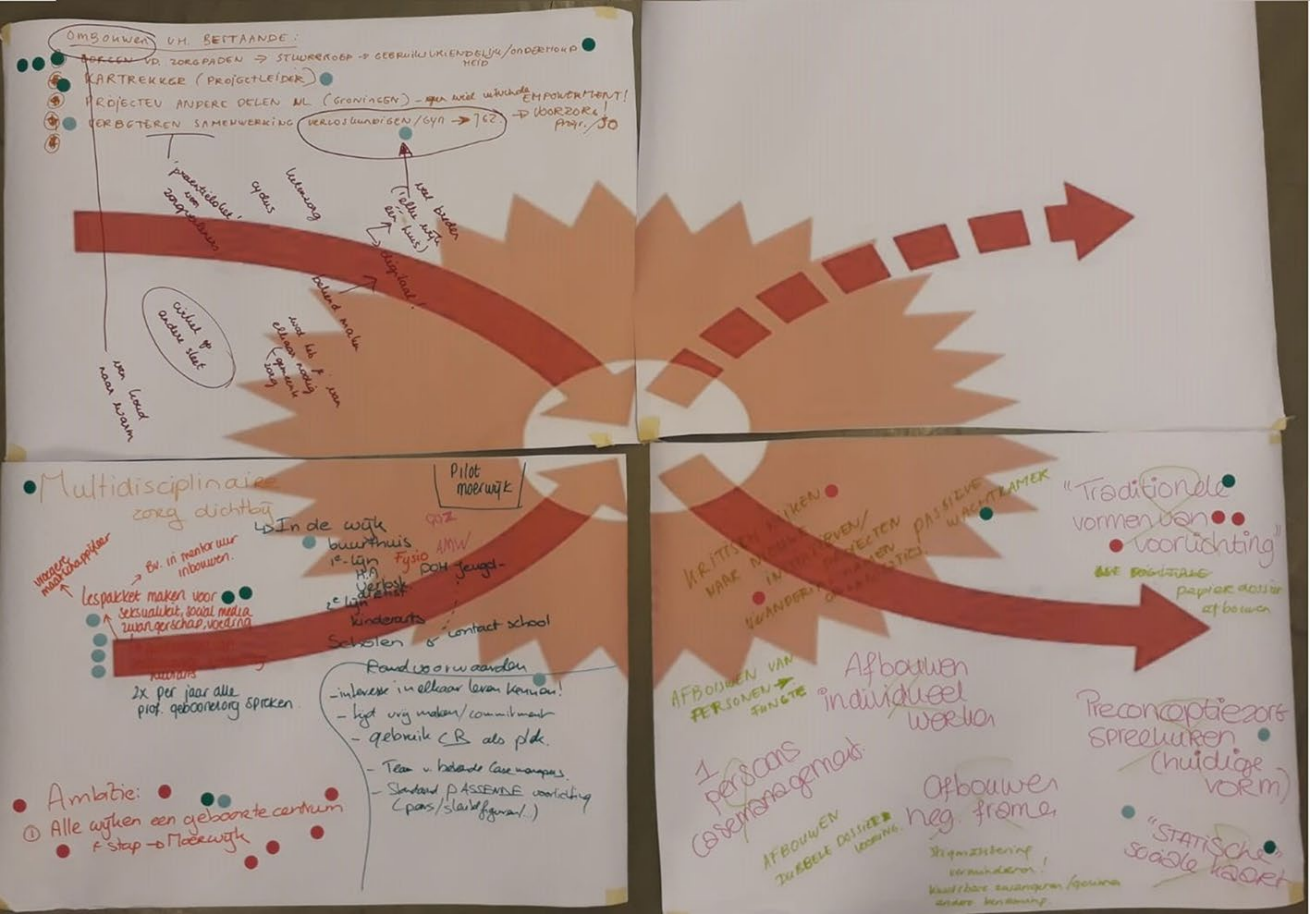
Output from the ‘Healthy Pregnancy Project’: participants identified transition dynamics and their own roles in the broader transition on a large X-curve printout (in Dutch). Photo: Derk Loorbach

Identifying potential measures and tools, governance mechanisms, and pathways to impact was one of the key goals of the ‘*healthy pregnancy project*’. This was challenging, as the project featured a particularly diverse yet disconnected group of professionals from the medical and social and public health care sectors. Here, the X-curve offered a valuable tool for a structured approach to formulating a local action agenda for tackling health inequities. The X-curve enabled joint reflections on the system in transition and which transition dynamics needed to be strengthened. Following this step, participants were asked to prioritise the identified actions through voting. All participants indicated which actions should be taken in the short term, mid-term, and long term. These resulted in a joint X-curve, with possible future steps and actions, which were then used to formulate local action agendas. Here, the visual representation of the change that the X-curve offers facilitated the design process of operational activities in relation to their tactical and strategic dimensions (see Fig. 3).

### Overcoming challenges in the use of the X-curve

While the X-curve is a useful framework for understanding and guiding sustainability transitions, there are a few challenges that surface across the cases. In some cases, these challenges appear functional, as they enable participants to confront deeply rooted beliefs, while in other cases, they demand strong facilitation capacities to guide the participants through the process.

#### Understanding chaos, destabilisation, and breakdown

In discussions, the notions of chaos, destabilisation, and breakdown were most often found to be difficult for participants to understand. These notions were often interpreted as inherently negative and something that should be avoided at all costs. However, we perceive this as an opening to confront this thinking and incite a meaningful discussion about the role of actors in these dynamics, leading to gaining the perspective that potentially destabilising or chaotic events are an inherent part of transitions. In other words, they can be seen as a sign of the need for transition (or a sign of the movement towards transition), rather than treating these events as incidents that can or should be resolved through optimisation. While chaos is often considered a window of opportunity for innovation (Holling 2001), chaos or crisis can also signal the acceleration of a niche practice that results in a period of uncertainty, leading to difficult but unavoidable questions on how to handle the sometimes-chaotic changes brought about by the acceleration of an alternative practice (Kauffman 1996; Kanger and Schot 2019). In this context, chaos can be a window of opportunity for change and growth that was formerly unfeasible in the existing regime context. This was particularly visible in the ‘*university minor*’ and the ‘*ministry advisory work*’. In both cases, friction regarding the understanding of chaos was resolved through open discussion with explicit reference to the interaction between breakdown and build-up dynamics. Facilitators of the ‘*healthy pregnancy project*’ noted that participants found it easier to think of build-up or redesign dynamics, rather than of dynamics of breakdown or chaos. In this case, the challenge was overcome by returning to what was identified as undesirable and unsustainable. Across all cases, discussion on chaos, breakdown, and destabilisation proved a challenging but necessary endeavour.

#### Thinking in terms of transition dynamics

Second, throughout the workshops of the ‘*ministry advisory work*’, civil servants tended to think mainly about policy implications. However, the use of the X-curve in the workshops illuminates that there are also organisational and even personal implications. This means that questions may arise about in which X-curve dynamic organisations or people can thrive and what new competencies are needed if this would change. This in turn leads to a discussion on whether civil servants see a role or responsibility for their own organisation and themselves, while faced with this need for intervention. A similar process was visible in the ‘*healthy pregnancy project*’, where the participating local policymakers preferred to think in terms of policies and regulations to guide change, while professionals from the medical sector preferred to discuss more applied medical solutions. In general, participants preferred talking about tangible activities instead of thinking about broader challenges and system features. That is, they recounted experiences with patients/clients from their everyday work life without translating them into broader systemic patterns. Here, the X-curve helped to provide a broader, long-term perspective and have participants reflect upon how the daily activities relate not only to longer-term change but also to other daily activities elsewhere in the system. For example, efforts to develop and diffuse new practices are hampered by efforts at the regime level to strengthen dominant approaches.

#### Concealing elements and complexity

Part of the X-curve’s strength is its ability to reveal and portray complex transition dynamics that would otherwise be difficult to recognise or interpret. However, the X-curve is in no way a comprehensive framework that manages to portray all relevant dynamics. By revealing some transition dynamics, other key determining elements of societal transitions have been concealed. This has, for example, been the case for exogenous trends, boundaries of the transition dynamics, and lack of a time dimension. In most cases, we consider the discussion regarding these concealing elements—that sooner or later always pop-up—as part of the social learning process. Moreover, the X-curve has proven to be flexible in its application and allows for the inclusion of complementary frameworks and concepts that can reveal these aspects.

For example, in the ‘*university minor*’, the absence of exogenous trends (i.e., landscape developments) in the X-curve framework proved an obstacle to understanding the system in transition and needed further elaboration. Considering the influence of such macro trends (such as the trend of digitalisation, or a disruptive global event such as the COVID-19 pandemic) is crucial in defining long-term societal trajectories and is therefore likely to affect the sector under study. Given their importance, students were encouraged to adapt the framework by capturing these landscape dynamics outside of the X-curve. In other cases, we have found that when mapping transition dynamics in a group setting, a lack of clear boundaries for what defines the different transition dynamics presents difficulties or incongruencies among actors, i.e., when can an event be understood as a sign of acceleration and when does it start to be a sign of emergence? As a clear operationalisation of what distinguishes the diverse dynamics from one another is missing, it has proven difficult for participants to distinguish one stage definitively from another. Another challenge for interpretation has been the visually implied linear time dimension and correlation between the occurrence of dynamics reciprocal to each other (e.g., institutionalisation and breakdown coinciding, appearing like a match). However, it is up to the participant’s interpretation of transition dynamics whether these matches in the figure are representative of their specific context. Completely different X-curve figures could be drawn, showing transition dynamics operating on incongruent patterns. Here, we consider it useful to explore and expand on transition cases that display these different interaction patterns; these can be used to illustrate the plural pathways that might potentially emerge to participants aiming to make sense of these dynamics of change.

## Discussion

The presented X-curve framework aims to further the understanding of the dynamics of transitions-in-the-making, as well as to support societal actors in navigating these dynamics. The X-curve brings the explicit need to understand destabilisation, breakdown, and phase-out of unsustainable regimes to the fore and frames this to provide space for alternative sustainable practices to scale up and institutionalise promising alternatives. As such, it forces policymakers or other applicants to think beyond innovation policy alone; for example, a sustainable energy transition is both about the phase-out and managed decline of fossil energy and the emergence and mainstreaming of renewables. The X-curve thus supports a more reflexive governance perspective by capturing the interaction between build-up and breakdown dynamics. This perspective allows for a temporary and contextual estimation of the ‘state’ of transition, its direction, and speed. This forms a basis for strategy development aimed at anticipating subsequent phases, responding to (un)desired dynamics, and exploring possible futures. More broadly, it could be argued that the focus on regime destabilisation and phase-out is the logical result of a transition accelerating, and the increasing build-up dynamics would eventually clash with the regime leading to destabilisation and sooner or later leading to a more fundamental discussion about the potential phase-out of established practices. As a result, our thinking on (the need for) dealing with these emerging transition dynamics co-evolved with what was happening in the practice of our dialogues with stakeholders.

We consider the ability to support understanding and navigation of transition dynamics to be the result of the X-curve framework’s main characteristics, which are (1) its simplicity, (2) the versatility allowing the embrace of plurality, and (3) the framing of chaos as inherent to transitions. First, it visually captures a transition narrative in an intuitive and simple manner, which upon closer inspection reveals the complex underlying dynamics. Here, revealing some aspects of a situation, while purposefully concealing others, allows to draw attention to particular dynamics within sustainability transitions (Ison et al. 2013). This simplicity helps to cultivate the understanding of the interplay between build-up and breakdown dynamics that can result in both reinforcing and counteracting change dynamics; i.e., experimentation might be retained by optimisation, but experimentation might also lead to destabilisation. Second, in this paper, we have illustrated the versatility of the X-curve’s use across diverse sectors (e.g., food, mobility, circular economy, or public health) and for self-reflection among variously empowered actors (e.g., policymakers and civil servants, medical practitioners, or students). This versatility in use and interpretation is important as it allows for an understanding of plural plausible transition pathways (Stirling 2008, 2011; Pel et al. 2020). Lastly, across cases, the X-curve showed its ability to reframe chaos from something that needs to be avoided at all costs towards something that is inherent to transitions and therefore needs to be faced and/or addressed (Loorbach et al. 2017; Walker et al. 2020). This allows actors to become more reflexive about their own role in relation to these dynamics, enabling them to adapt behaviour to it and seek out collaborations where necessary or beneficial.

## Conclusion

The X-curve is a conceptual framework that offers guidance in understanding and developing governance practices for sustainability transitions. It was developed to support diverse societal actors in understanding the dynamics of societal transitions, where dynamics of breakdown and decline are becoming more obvious and visible. We stress the importance of frameworks that address these dynamics more explicitly as people tend to focus on novelty over thinking about subtractive changes when trying to solve problems.

The X-curve builds on existing frameworks, which traditionally have a stronger emphasis on build-up dynamics, and adds insights from research exploring breakdown and phase-out dynamics. As such, the framework characterises transition dynamics as a combination of build-up, breakdown, and their interactions. Besides synthesising these academic insights, we demonstrated how, when used in sustainability practice, it proves to be an actionable framework. Using three cases, we illustrated the X-curve’s main strength as a framework that can support groups of people to develop a shared understanding of the dynamics of both build-up and breakdown in transitions-in-the-making. This helps them reflect upon their roles, potential influence, and the needed capacities for the desired transition. Moreover, we find the framework can serve the key types of knowledge identified for transformation to sustainability, namely, system, normative, and transformational knowledge.

The X-curve can be a starting point for navigating long-term change by facilitating a shared understanding and reflexivity. Nonetheless, there are some challenges in terms of dealing with mixed understanding of concepts offered by the framework—such as chaos and destabilisation; difficulties with thinking in terms of transition dynamics that have no clear boundaries and parameters; and the fact that, by revealing *some* transition dynamics, the X-curve also conceals others. Even so, we have shown that it is exactly its simplicity, versatility to use it in various sectors, and ability to reframe chaos that make the X-curve such a useful framework in making sense of present-day transition dynamics. With this paper we intended to articulate the development and illustrate the use of the X-curve in a way that others can use it, reflect on it, build on it, and judge its value for action research on sustainability transitions themselves.

## Supplementary Information

Below is the link to the electronic supplementary material.

Supplementary file1 (PDF 139 KB)

## Footnotes

1 The starting point for the multi-level perspective was the notion that socio-technical systems (such as a mobility system or energy system) are marked by dominant socio-technical structures (e.g., technologies, institutions, markets, infrastructures, cultural preferences) that stabilise embedded practices—the ‘regime’ (Geels 2005). Regimes are considered to provide social stability and allow for efficient, rapid adjustments if the external environment changes incrementally. However, this inherent stability comes at the cost of lock-ins and path-dependencies that hamper transformative change (Rip and Kemp 1998).

2 The multi-phase concept (also known as the ‘S-curve’) described the pattern of disruptive and non-linear transformative change emerging from so-called ‘niches’ which provide fundamental alternatives to an existing regime. Resembling Rogers’ innovation-diffusion curve (Rogers 1995; Rotmans et al. 2001), it describes how a longer period of niche predevelopment leads to emerging and accelerating alternatives that then break through and mainstream. It emphasizes the non-linearity and transformative nature of transitions, as well as an underlying pattern of ‘evolutionary revolution’ (Rotmans et al. 2001).

3 Key to DRIFT’s advisory, education, and research approach is the understanding that co-creation processes between science and societal actors are essential for the generation of action-oriented knowledge (Jasanoff 2004; Stirling 2008; Kirchhoff et al. 2013; Wittmayer et al. 2014; Clark et al. 2016; Fazey et al. 2018; Schneider et al. 2019; Caniglia et al. 2020). That means new knowledge and (transformative) capacities of involved actors emerge from an entangled process that combines action(-research) and capacity building through the co-creation and transdisciplinary involvement of diverse societal actors (Lang et al. 2012).

4 Transition management emerged as a governance approach for sustainable development that builds on transition theory insights, aiming at ‘long-term planning through small steps’ (Loorbach 2010; Rotmans and Loorbach 2010, p. 140). The transition management cycle is intended for use in an applied setting and leans on strategic, tactical, operational, and reflexive activities (Loorbach 2010).

5 The themes the Dutch Ministry of Infrastructure and the Environment was interested in were energy transition, smart and green mobility, circular economy, liveable and accessible cities, and climate change adaptation (Lodder et al. 2017).
