# Supplementary material for: An actionable understanding of societal transitions: the X-curve framework
Source: Sustain Sci. 2022 Jan 18;17(3):1009–21. doi: 10.1007/s11625-021-01084-w (PMC8764493; doi:10.1007/s11625-021-01084-w)
Supplement: Supplementary file 1 — Supplementary file1 (PDF 139 KB) [file 11625_2021_1084_MOESM1_ESM.pdf]

Supplementary Material

In-depth description of the case 'Ministry advisory work'

The Dutch Ministry of Infrastructure and Water (hereafter Ministry) commissioned an analysis of the 'state of transition' of the policy domains that are its responsibility (mobility, circular economy, and climate adaptation in the built environment) and through that identify potential governance interventions. This advisory project entailed a combination of desktop analysis, in-depth semi-structured expert interviews and several workshops co-organised with civil servants. These workshops had two main objectives: First, for civil servants to internalise thinking in dynamics of build-up and break-down by using the x-curve framework, as this would benefit their understanding and uptake of the analysis findings. Second, for data collection, as the civil servants provide valuable insights about transition dynamics and the policy context. Indeed, the workshops were vital for the quality of the analysis and the legitimacy of the results and policy recommendations of the commissioned analysis.

Use of the framework

The Ministry's civil servants were introduced to transition thinking and the X-curve framework through a short explanatory lecture at the start of the workshop. After, through discussions with the civil servants, attention shifted to the relevant policy domains, such as mobility. This entailed discussing the boundaries of the relevant system (e.g., is aviation included or excluded?) and their future vision of the system. These discussions were crucial, as they revealed underlying assumptions about a desired future and allowed to make radical visions more explicit.

The next step in the workshop was for civil servants to use the framework themselves. In an exercise in small groups (5 to 10 persons), the civil servants were asked to identify examples of transition dynamics within the established boundaries and with relation to the future vision. Aided by a large X-curve print-out and an explanatory table detailing the transition dynamics (table 1 in paper), they noted transitions dynamics on post-its and placed these on the X-curve print-out. Following, the groups reflected on the created post-its X-curve and discussed what stood out, what was expected and surprising.

Next, the workshop aimed at inducing an action-perspective among the civil servants. Given the identified transition dynamics, the civil servants were asked to identify where the Ministry could intervene to 1) enable the build-up of new; 2) scale-up promising practices; and 3) convert existing or 4) phase-out unwanted practices, ideas, and structures. To capture these alongside the identified dynamics, a distinct colour post-it was used in a second round when additional post-its were placed on the x-curve framework print-out. This was concluded by a plenary discussion, in which discussants were asked to emphasize the role of the Ministry in steering transitions, rather than to discuss the identification of transition dynamics.

The data from the desk research, interviews, and the output of these workshops were analysed through the X-curve framework to get an assessment of the state of transition. For each of the identified transition dynamics (e.g., experimenting or destabilization), examples were added with a brief explanation why these examples were interpreted as an expression of this particular dynamic (see table 2 in main paper).

Challenges in the use of the framework

During discussions, the civil servants found that the notions of chaos, destabilization and break-down were sometimes difficult to understand and contextualize. They were often interpreted as inherently negative and something the Ministry should avoid at all costs. In other words, these dynamics were not (yet) identified as inherent to deep structural system change and related to contexts of de-institutionalization with high degrees of uncertainty. The discussions facilitated the co-creation of an understanding that these dynamics are inherent to transitions, which supported the interpretation of potentially destabilizing or chaotic events as a part of transitions. Rather than treating these events as incidents that can be resolved through optimization, they were instead seen signs of the need for transition. Finally, throughout the workshops civil servants had the tendency to think in policy implications. However, the use of the X-curve in the workshops illuminates that there are also organizational and even personal implications. Such as, in which dynamics of the X-curve do organizations or people thrive, and what new competencies are needed if this would change. This in turn leads to recognition and discussion on whether civil servants see a role or responsibility for their own organization and themselves, while faced with this need for intervention.

Dynamics of change identified & capacities gained

The use of the x-curve framework facilitated the joint identification of different activities and practices that can currently be observed in the system through the eyes of civil servants. Additionally, this helped validate the data collected via desktop-research and interviews, thereby contribution to an enhanced understanding and informed discussion about transition dynamics. This included a rough assessment of what transition dynamics are most relevant and visible in the current system, which served as a starting point for social discussion about the state of transition.

Notable was that after civil servants identified intervention points, discussions became more reflexive by questioning whether their role as Ministry in this transition. For instance, they reflected whether their current focus on particular dynamics and their skillset was right, but also whether the competencies and powers of the Ministry were appropriate to navigate and steer the current transition. Most remarkable was that using the X-curve, dynamics such as break-down and phase-out become a core topic of discussion, especially in relation to upward, innovation, dynamics. This is paramount, as the Ministry has a persistent innovation bias that usually led to a one-sided focus on innovation and improvement (ref). As such, use of the X-curve supported civil servants in uncovering common blind spots, such as existing solutions in need of more support and the need to phase out existing unsustainable practices.

In-depth description of the case ‘University minor’

At the Erasmus University Rotterdam, the X-curve framework has been applied in the context of university-level education. In the course ‘New Economic Thinking & Social Entrepreneurship’ bachelor students are trained in reflecting about alternative economic models, transition theory and societal impact. The design of the course is interactive and requires students to team up with start-ups or social enterprises and apply lessons from the course to support these start-ups and social enterprises in meeting their sustainability-oriented missions.

Use of the framework

The students are introduced to the framework during an introductory lecture, which offers an overview of transition theory and its key models, including the x-curve framework. As such, concepts and assumptions underlying the framework are explained and illustrations of the framework’s applications are shown to indicate how it can be used to analyse the transition dynamics in different sectors or systems. Building on this lecture, student groups experience uses of the framework through an assignment aimed at applying the X-curve for analysis and reflection on change dynamics relevant to their start-ups and social enterprises. For this, groups receive a large print out of the x-curve framework, along with steps and questions to guide the students through use of the X-curve.

The first step of the assignment is to define the sector(s) or subsystems in which their start-up or social enterprise operates and to conduct a rough system analysis to identify current (un)sustainable dynamics, challenges embedded in the system, and key system actors.

In the second step, the students zoom in on the transition dynamics relevant to the start-up or social enterprise’ sector alone. They do so by answering the guiding questions and visualizing the answers on the large x-curve print-out. Questions are connected to a certain dynamic of the x-curve. Such as a question aimed at identifying dynamics of acceleration: “*Which emerging innovative/alternative practices are gaining traction?*” or to identify dynamics of destabilisation: “*What are examples of destabilization or developments that challenge the current status quo in the sector?*” From the extensive list of questions, students pick three expand on in the assignment.

This marks the starts of the third, deliberative and analytical, step of the assignment in which the students get one week to further unpack and analyse the sectors under study. Students are asked to note answers to the chosen three questions on post-its, which can be stuck on the large print out of the x-curve received earlier. The following lecture, students are invited to share insights from their group analysis, which are plenary discussed and reflected on.

Dynamics of change identified & capacities gained

Using the X-curve, students’ understanding, and descriptions of sector characteristics shift from a static understanding towards more dynamic understandings of system change and its immanent facets. In addition, by linking this newly gained dynamic picture of the sector with the enterprise they teamed up with, they can identify their alternative practices (e.g., novel business model, socio-technological innovation) and capacities of different actors to intervene and navigate. Based on this, students are

encouraged to develop ideas on how the teamed-up enterprise can adapt or navigate this context, and which policy interventions or additional governance structures would be necessary to support desired dynamics. However, it is the third reflexive step, that the X-curve framework demonstrates its capacity building function: Students can use the framework in a strategic manner, by generating ideas about how actors (e.g., enterprises, policy makers) may adapt or navigate the current and possible future sector dynamics.

Challenges in the use of the framework

The uptake of learnings using the X-curve by bachelor student is promising, however there are some observed challenges and limitations. The overall concept of the X-curve framework was well understood, however some of the specific dynamics described within the framework demanded further explanation. For example, the phase of 'chaos' evoked several critical questions and potential misunderstandings during the introduction lecture. The feedback of students suggested that 'chaos' was mostly connotated as a 'negative' dynamic, associated with discomfort and ideally should be averted. As such, chaos was not (yet) interpreted as a dynamic inherent to deep structural system change that is related to de-institutionalization and high degrees of uncertainty. This led to students reflecting on the relevant question: "*Can there be a transition without chaos?*" To better accommodate these reflections reference to evolutionary economics and theories of creative destruction are in order, to reframe the immediate assumptions students have with the notion chaos.

An additional challenge as for students to connect single enterprises to broader sector dynamics, which relates to the absence of exogenous trends (i.e., landscape developments) in the X-curve framework. Their influence is crucial in defining long-term societal trends and are likely to affect the sector under study. Given their importance, students were encouraged to adapt the framework by capturing these dynamics outside of the X-curve. While this is a practical solution to visualizing and capturing external factors, it would be beneficial to offer additional reflections or knowledges about the embedding and interconnectedness of individual actors within the broader system.

In-depth description of the case 'Healthy pregnancy project'

The Healthy Pregnancy 4 All-3 (HP4All-3) research project (2018-2021) aims to identify ways to address perinatal (i.e., the period around the time of birth) health inequities in the Netherlands. Health inequities that are present at birth affect individuals' health and socioeconomic outcomes across the entire life course. As health at birth is not only influenced by medical risk factors but also by non-medical risk factors, such as a low educational level, psychosocial problems, lack of social support, and neighbourhood deprivation, a comprehensive cross-sectoral care approach is needed, in which professionals from the medical sector collaborate with professionals from the social and public health care sectors. The dynamics and mechanisms that might lead to a 'sustainability transition' towards such a cross-sectoral approach to perinatal health have yet to be identified. The HP4All-3 programme is a collaboration between the Erasmus MC, the Dutch Research Institute for Transitions (DRIFT), and the Dutch Centre of Expertise on Health Disparities (Pharos) (see Barsties et al., under review).

The research took place in six Dutch municipalities (i.e., Eemsdelta, Enschede, The Hague, Vlissingen, Landgraaf, and Heerlen) that all struggle with a high incidence of adverse perinatal health outcomes (i.e., preterm birth and small for gestational age) but vary with regards to the implementation of perinatal health into their approaches and policies concerning health inequities. After extensive desk research and in-depth semi-structured expert interviews (8 to 10 per municipality) with professionals from the relevant sectors, two interactive group sessions with the same target population were organized in each municipality (in total 12 sessions with 96 participants). The aim of these group sessions was twofold: 1) to discuss the urgency of the problem at stake and 2) to jointly develop a local action agenda, consisting of agreements to be made and actions to be taken.

Use of the framework

The X-curve was the central framework for the group sessions. After presenting the results of our desk research and interviews at the beginning of the first session, the participants discussed current problems and future systemic changes that are needed to overcome local perinatal health

Supplementary table 1 Interpretation of the transition dynamics in the Dutch approach to perinatal health inequities

Pattern	of build-up	Pattern	of break-down
Experimentation	School teaching programmes on topics such as sexuality, pregnancy, healthy food, etc.	*Optimisation*	Clear care pathways; mapping professional networks in a less static way (digital networking); knowing the needs of the target population; clear protocols for referring patients to other organizations
Acceleration	Novel practices like 'Centring pregnancy,' 'Centring parenting'; peer mentors and educators for parents (to-be) living in precarious conditions	*Destabilisation*	Diverse ways of financing health care policies by insurance companies; customized health care services (less bureaucracy)
Emergence	Neighbourhood health care hubs which also offer perinatal health care, meetings, and collaborations between professionals from the medical and social sectors	*Chaos*	Free anti-conception for men and women
Institutionalisation	Building local coalitions with different stakeholders; structural funds for local basic facilities; easily accessible facilities and personal way of communicating between care professionals and their patients	*Breakdown*	Stigmatizing 'vulnerable pregnant women'; less medicalisation of pregnancy; less pillarized and sectoral ways of financing and collaborating
Stabilisation	Central registration of parents (to-be); early signalling of at-risk women and families	*Phase-out*	Traditional ways of counselling; individualistic ways of working

inequities in small groups. This discussion resulted in guiding principles. This was a preparatory step for the group exercise in the second session, in which participants were asked to identify tangible actions to realize these guiding principles. Participants were assigned to three small groups discussing 1) patterns of build-up, 2) patterns of break-down, and 3) patterns of continuation. Back in plenary, these three patterns were put together with the guiding principles that were discussed during the first session. This resulted in a complete X-curve. Next, participants were asked to prioritise the identified actions. By voting with stickers, the participants indicated which actions should be taken on the short-term (within one year), mid-term (one to five years) and long-term (more than 5 years). Together, these exercises resulted in six X-curves (one per municipality) with probable future scenarios and actions – divided into break-down, build-up and continuation. After finalisation of the group sessions, the X-Curves were used to formulate local action-agendas.

Challenges in the use of the framework

There were several challenges when working with the X-curve during the group sessions. First, not all participants found the X-curve comprehensible because of its level of abstraction. It was challenging to discuss the X-curve with participants from different experiential and professional backgrounds. For example, professionals working in the medical sector (i.e., midwives or general practitioners) tend to prefer practical solutions over abstract reasoning, while professionals working for the municipal government (i.e., civil servants or council members) are used to think in abstract policy terms.

Second, participants preferred talking about tangible activities instead of thinking in broader challenges and system features. That is, they were recounting experiences with patients/clients from their everyday work life without translating them to broader systemic patterns. Third, participants had difficulties thinking in terms of patterns of break-down. It was much easier to think of building up new activities or rebuilding existing ones. Although, they were able to voice what was currently undesirable and unsustainable, they found it hard to connect this to tangible activities and practices. However, many participants also complained about being too busy and being tired of new projects being initiated all the time instead of making best practices more durable. Breaking down unsustainable features and activities proved to be a necessary but challenging endeavour.

Dynamics of change identified & capacities gained

After some initial difficulties in working with the X-curve, participants valued the newly gained common language

and vocabulary. Especially in a professionally diverse setting as the HP4All-3 group sessions, the X-curve can help analysing current problems and envisioning the future one wants to work towards. Yet, there should be enough time to explain in detail the concept of and ideas behind the X-curve. One could think of different X-curve terminologies that can be used with people from various professional contexts as well as with different educational and experiential backgrounds. This could broaden the usability of the X-curve and make it accessible to more people, irrespective of their educational and professional background. The X-curve also offered a valuable tool for a structured and multidimensional approach to formulating a local action-agenda for tackling health inequities. As a visual representation of change, the X-curve facilitates the design process of operational activities in relation to their tactical and strategic dimensions. Finally, the X-curve enables participants/discussants to understand the dynamics of change, in terms of chronology and interdependency of build-up, break-down and continuation.
